# Comparison of revascularization with conservative medical treatment in maintenance dialysis patient with coronary artery disease: a systemic review and meta-analysis

**DOI:** 10.3389/fcvm.2023.1143895

**Published:** 2023-04-17

**Authors:** Ling Zheng, Xiang Wang, Yu-cheng Zhong

**Affiliations:** ^1^ Department of Cardiology, Union Hospital, Huazhong University of Science and Technology, Wuhan, China; ^2^ Department of Cardiovascular Surgery, Union Hospital, Huazhong University of Science and Technology, Wuhan, China

**Keywords:** revascularization, conservative treatment, dialysis, coronary artery disease, meta-analysis

## Abstract

**Background:**

The primary cause of death among maintenance dialysis patients is coronary artery disease (CAD). However, the best treatment plan has not yet been identified.

**Methods:**

The relevant articles were retrieved from various online databases and references from their inception to October 12, 2022. The studies that compared revascularization [percutaneous coronary intervention (PCI) or coronary artery bypass grafting (CABG)] with medical treatment (MT) among maintenance dialysis patients with CAD were selected. The outcomes evaluated were long-term (with a follow-up of at least 1 year) all-cause mortality, long-term cardiac mortality, and the incidence rate of bleeding events. Bleeding events are defined according to TIMI hemorrhage criteria: (1) major hemorrhage, intracranial hemorrhage or clinically visible hemorrhage (including imaging diagnosis) with decrease of hemoglobin concentration ≥5 g/dl; (2) minor hemorrhage, clinically visible bleeding (including imaging diagnosis) with a drop in hemoglobin of 3–5 g/dl; (3) minimal hemorrhage, clinically visible bleeding with hemoglobin drop <3 g/dl. In addition, revascularization strategy, CAD type, and the number of diseased vessels were considered in subgroup analyses.

**Results:**

A total of eight studies with 1,685 patients were selected for this meta-analysis. The current findings suggested that revascularization was associated with low long-term all-cause mortality and long-term cardiac mortality but a similar incidence rate of bleeding events compared to MT. However, subgroup analyses indicated that PCI is linked to decreased long-term all-cause mortality compared to MT but CABG did not significantly differ from MT in terms of long-term all-cause mortality. Revascularization also showed lower long-term all-cause mortality compared to MT among patients with stable CAD, single-vessel disease, and multivessel disease but did not reduce long-term all-cause mortality among patients with ACS.

**Conclusion:**

Long-term all-cause mortality and long-term cardiac mortality were reduced by revascularization in comparison to MT alone in patients undergoing dialysis. Larger randomized studies are needed to confirm the conclusion of this meta-analysis.

## 1. Introduction

CAD remains the primary cause of death in patients with chronic kidney disease including maintenance dialysis patients (2). A study showed that heart disease is the leading cause of death in maintenance dialysis patients, accounting for 44% of all-cause mortality. Acute myocardial infarction (AMI) is responsible for about 20% of cardiac deaths (3). The age-adjusted cardiovascular mortality of maintenance dialysis patients is 10–20-fold relative to patients without CKD (4). Despite the high mortality risk of CAD in maintenance dialysis patients, the optimal treatment strategy is yet unknown, and the argument is whether revascularization therapy is superior to conservative MT.

Nonetheless, the most randomized clinical trials (RCTs) in the cardiovascular field have either removed maintenance dialysis or included too few studies for a convincing assessment of treatment advantages (5–8). In addition, maintenance dialysis patients regularly display an “oligo-symptomatic” presentation of CAD (9), and the coronary artery has severe stenosis and calcification when symptoms appear, which increases the challenges and risks of surgery, especially PCI. Therefore, these high-risk patients in our clinical practice are undertreated with revascularization therapy due to perioperative complications and bleeding events (10). Moreover, there are no unified guidelines to standardize the management and treatment of such special groups. Some previous observational investigations supported revascularization (11), but a recent RCT, ISCHEMIA-CKD study (12), indicated that revascularization therapy less effective than the conservative MT for end-stage renal disease (ESRD) with stable CAD. Whether such patients should opt for an invasive strategy or drug-conservative treatment has been controversial in the academic community. Therefore, the present study aimed to identify the best treatment strategy for CAD in maintenance dialysis patients by conducting a meta-analysis of eligible research.

## 2. Methods

### 2.1. Inclusion and exclusion criteria

The following criteria had to be fulfilled by eligible studies: (1) included maintenance dialysis (for at least 3 months) patients with CAD (had ≥50% diameter stenosis in coronary artery or diagnosed with acute coronary syndrome, ACS); (2) compared revascularization (PCI or CABG) with MT alone; (3) reported one or more of the following outcomes: long-term all-cause mortality, long-term cardiac mortality, and the incidence rate of bleeding events (12); (4) RCTs or observational studies. The exclusion criteria were as follows: (1) the number of patients was <50; (2) the duration of follow-up was <1 year; (3) failure to report any of the above outcomes; (4) articles not in Chinese or English; (5) registries with overlapping patients.

### 2.2. Search strategy and data extraction assessment

We searched PubMed, the Cochrane Library database, Embase, China National Knowledge Infrastructure (CNKI), Wanfang Patent Database (WFPD), China Science and Technology Journal Database, Chinese Clinical Trial Registry (ChiCTR), and Clinical Trials from inception to October 12, 2022, using the following keywords and MeSH terms: renal dialysis; kidney failure, chronic; myocardial revascularization; coronary artery bypass; percutaneous coronary intervention; conservative treatment; drug therapy. In addition, we manually searched through all the references of important reviews to identify any eligible studies. Two authors (Ling Zheng and Yu-cheng Zhong) reviewed eligible studies and extracted patient data. The study data included country of study, year of study publication, study design, number of patients included, follow-up duration, and type of revascularization. The patient data included gender, age, duration of dialysis, the percentage of patients who have stable CAD, multivessel disease, and diabetes. Two researchers searched the databases, retrieved the relevant articles, and extracted the data. Any discrepancies were resolved by discussion.

### 2.3. Quality assessment

RCT quality was assessed by Cochrane Collaboration's tool, and the quality of observational studies was evaluated using the Newcastle–Ottawa Scale.

### 2.4. Statistical analysis

RevMan 5.3 software was applied to pool the relative risk (RR) as an effect with a 95% confidence interval (CI), and Stata MP software version 16.0 performed sensitivity analysis. Heterogeneity among studies was assessed by estimating *I*^2^ statistic. We chose the M-H fixed-effects model to calculate the pooled effect when *I*^2^ was <50%, and M-H random-effects model was utilized when *I*^2^ was >50%, suggesting significant heterogeneity. Also, a sensitivity analysis was conducted to explore heterogeneity when it was high, and the stability of results was assessed with a “leave-one-out” approach. The publication bias was visually assessed by funnel plots.

## 3. Results

### 3.1. Study selection and quality assessment

**Figure 1:**
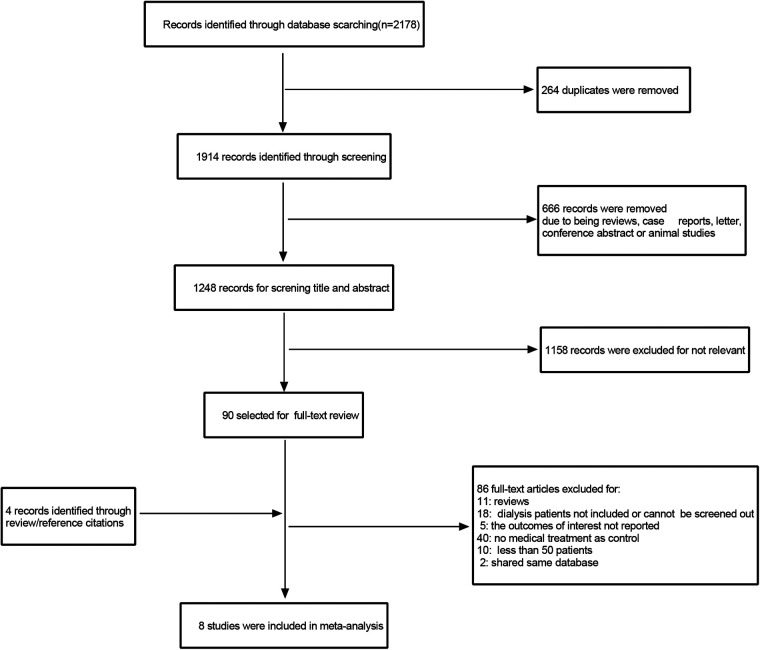
Study selection.

**Figure 2:**
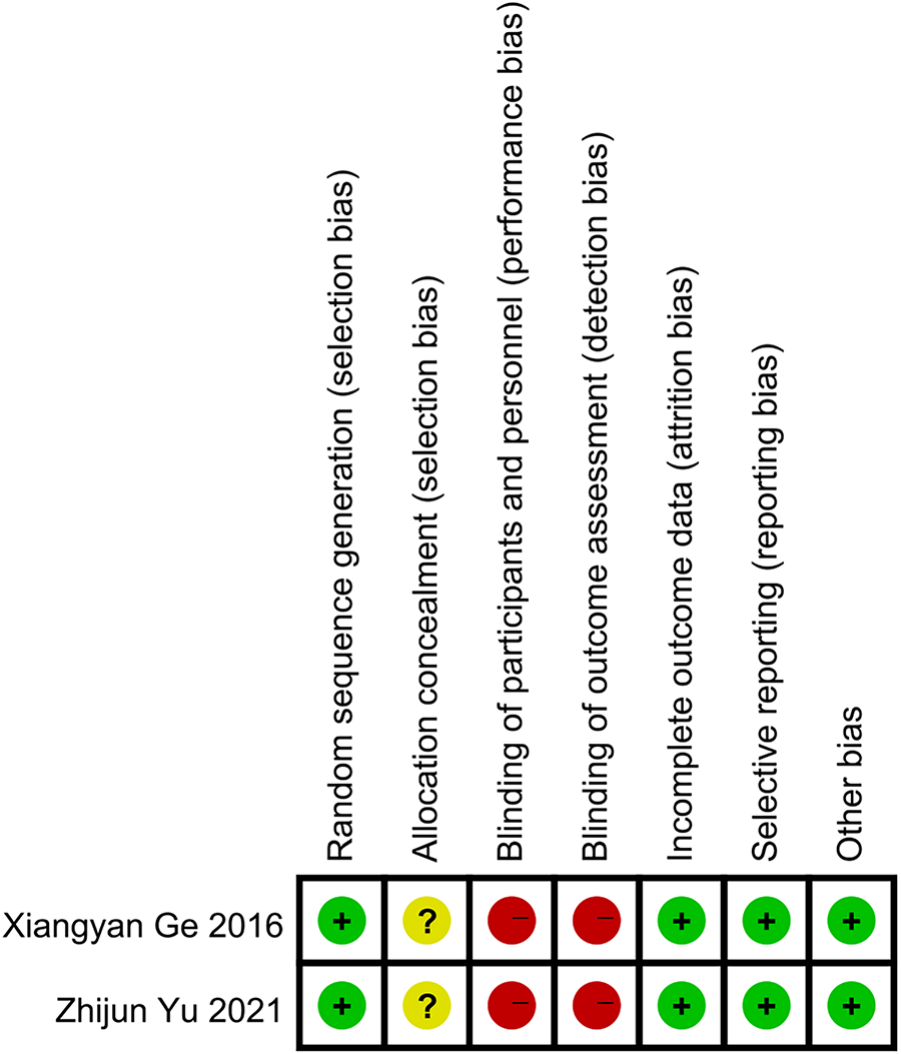
Risk of bias graph in the two RCTs.

**Figure 3:**
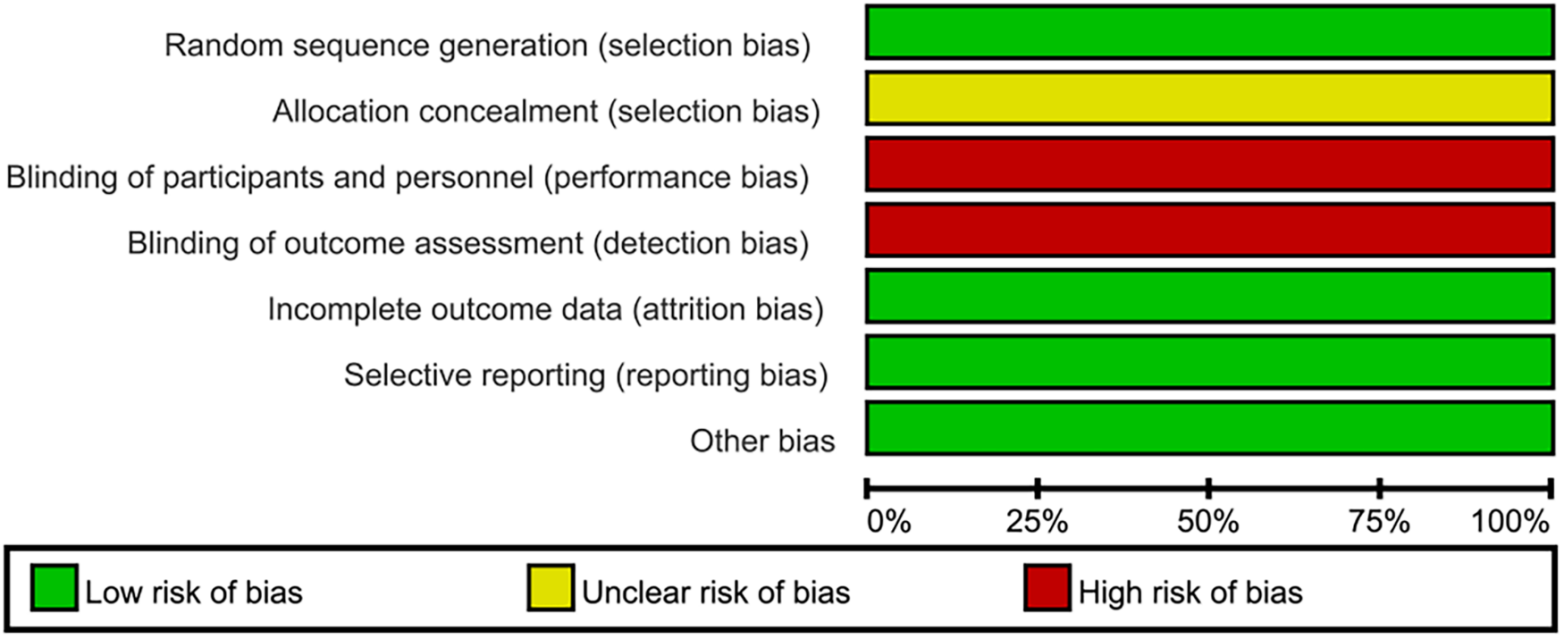
Risk of bias summary in the two RCTs.

A total of 2,178 related studies were retrieved from different online databases. After removing 264 duplicates, 1,914 unique records were screened, followed by assessing the titles and abstracts of 1,248 records. The full text of 90 articles was browsed after the exclusion of 1,158 irrelevant studies. In addition, four articles were retrieved manually through references and citations. Thus, 94 complete texts were evaluated for eligibility. Finally, eight studies (13–20) meeting criteria were included in the review and meta-analysis: two RCTs and six observational studies. The selection process is illustrated in Figure 1. The two RCTs had a low risk of bias, while the NOS values of the six observational studies ranged from 7 to 8, indicating a high quality (Figures 2, 3, Table 1).

**Table 1:** Quality assessment of NOS.

Study	Selection	Comparability	Outcome	Score
Chertow 2000 (13)	★★★	★★	★★	7
Yasuda 2006 (14)	★★★★	★	★★	8
Sakakibara 2011 (15)	★★★★	★★	★★	8
Yeates 2012 (16)	★★★★	★	★★	7
APPROACH 2018 (18)	★★★★	★★	★★	8
Jun-ting Zhang 2020 (19)	★★★★	★	★★	7

### 3.2. Study characteristics

Table 2 provides a summary of the studies' baseline characteristics. This meta-analysis included a total of 1,685 patients: 739 underwent revascularization and 946 received MT alone. The follow-up period was 1–8 years, and the median follow-up period was 1–3.6 years. In addition, most of the patients were males and elderly, and >50% had diabetes. Only two studies (Yasuda, 2006; Zhang, 2020) reported the number of vessels involved, and the data showed that multivessel disease is common in dialysis patients. The MT included antiplatelet agents, statins, ACEIs/ARBs, and β-blockers except one study (Chertow, 2000) which did not report the details of MT.

**Table 2:** Baseline characteristic.

Study	Country	Design	Follow-up (years)	Median follow-up time (years)	Types of Re	*N*	Age	Male, %	Duration of dialysis (years)	Stable CAD, %	MVD, %	Diabetes, %
Chertow 2000 (13)	USA	OS	1	NR	PCI and CABG	640	NR	59	NR	0	NR	52
Yasuda 2006 (14)	Japan	OS	5	3.25	PCI	134	63.3	64.2	5.31	64.9	66.4	57.4
Sakakibara 2011 (15)	Japan	OS	8	3.6	PCI	391	NR	NR	NR	NR	0	NR
Yeates 2012 (16)	Australia	OS	4.5	1	PCI and CABG	90	60.9	60.0	1.08	NR	NR	50
APPROACH 2018 (18)	Canada	OS	8	3.2	PCI and CABG	118	62.8	73.7	NR	100	NR	67
Xiang-yan Ge 2016 (17)	China	RCT	1	1	PCI	100	64.5	61.0	3.66	0	NR	32
Jun-ting Zhang 2020 (19)	China	OS	5	NR	PCI and CABG	100	59.8	50.0	7.26	NR	82	36
Zhi-jun Yu 2021 (20)	China	RCT	1	1	PCI	112	62.2	61.6	3.12	0	NR	24.1

MVD, multivessel disease; NR, not reported; *N*, number; USA, United States of America; OS, observational study; Re, revascularization.

### 3.3. Long-term all-cause mortality

**Figure 4:**
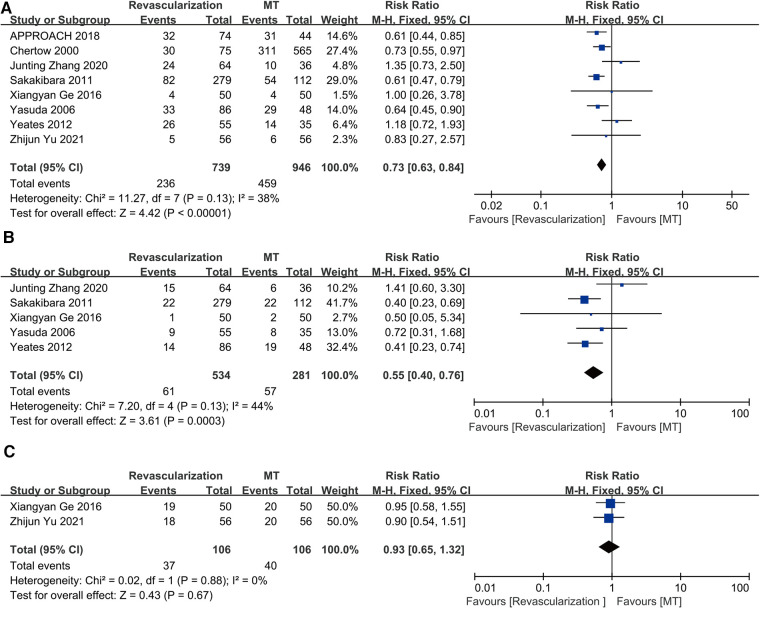
Revascularization vs. medical therapy: (**A**) long-term all-cause death; (**B**) long-term cardiac mortality; (**C**) the incidence rate of bleeding events. CABG, coronary artery bypass grafting; CI, confidence interval; MT, medical therapy; PCI, percutaneous coronary intervention.

Eight studies were combined for long-term all-cause death. The results showed that revascularization was linked to lower long-term all-cause mortality than MT (RR = 0.73, 95% CI = 0.63–0.84). The eight studies had a moderate degree of heterogeneity (*I*^2 ^= 38%, *P* = 0.13) (Figure 4A).

### 3.4. Long-term cardiac mortality

Cardiac death was assessed in five studies. The results showed that invasive therapy was linked to a decrease in long-term cardiac mortality (RR = 0.55, 95% CI = 0.40–0.76). The five studies showed only mild heterogeneity (*I*^2 ^= 44%, *P* = 0.13) (Figure 4B).

### 3.5. The incidence rate of bleeding events

Only two studies (Ge, 2016; Yu, 2021) reported the outcome of bleeding events. The revascularization strategy of these two studies was PCI, and the patients who underwent PCI were treated with dual antiplatelet therapy (DAPT) for 1 year. The patients in MT group were treated with a single antiplatelet agent (aspirin 100 mg). The number of major hemorrhage events, minor hemorrhage events and minimal hemorrhage events in the PCI and MT groups were 3, 1, 15 and 2, 1, 17, respectively, in the study of Ge et al. and the total number of bleeding events in the PCI and MT groups were 18 and 20, respectively, as presented by Yu et al. without the detail of bleeding events. An analysis of the two studies did not show any difference in the incidence rate of bleeding events between revascularization and MT (RR = 0.93, 95% CI = 0.65–1.32) (Figure 4C), and they showed no heterogeneity (*I*^2 ^= 0%, *P* = 0.88).

### 3.6. Subgroup analyses

#### 3.6.1. PCI vs. MT

**Figure 5:**
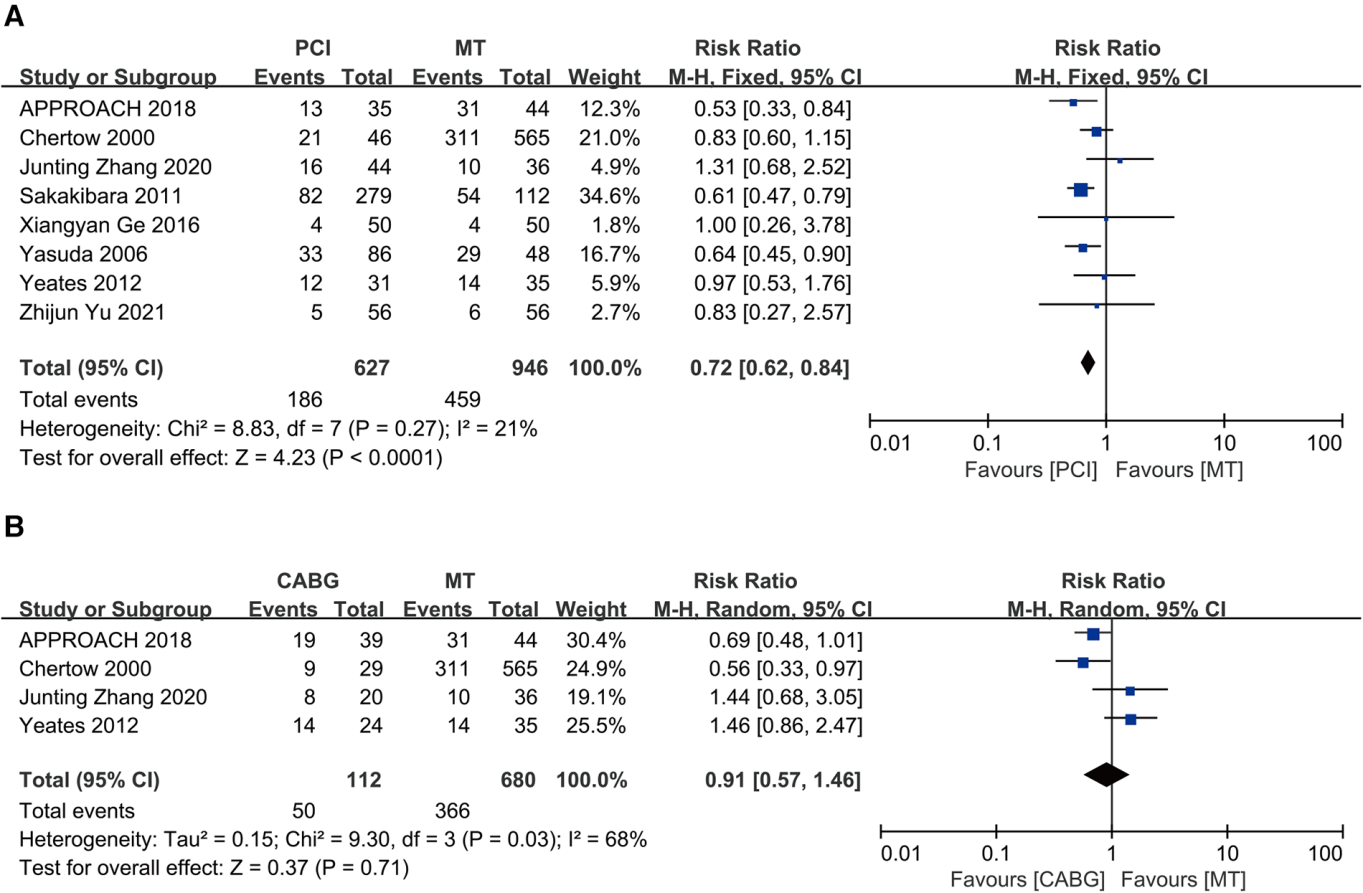
Long-term all-cause death: (**A**) PCI vs. MT; (**B**) CABG vs. MT; CABG, coronary artery bypass grafting; CI, confidence interval; MT, medical therapy; PCI, percutaneous coronary intervention.

A total of eight studies compared PCI to MT in maintenance dialysis patients with CAD, including 627 patients receiving PCI and 946 receiving MT. The results indicated that PCI is associated with low long-term all-cause mortality (RR = 0.72, 95% CI = 0.62–0.84), and the eight studies revealed mild heterogeneity (*I*^2 ^= 21%, *P* = 0.27) (Figure 5A).

#### 3.6.2. CABG vs. MT

Four studies compared CABG with MT among maintenance dialysis patients with CAD, including 112 patients receiving CABG and 680 receiving MT. The results showed similar long-term all-cause mortality between CABG and MT (RR = 0.91, 95% CI = 0.57–1.46) but significant heterogeneity (*I*^2 ^= 68%, *P* = 0.03) (Figure 5B).

#### 3.6.3. ACS

**Figure 6:**
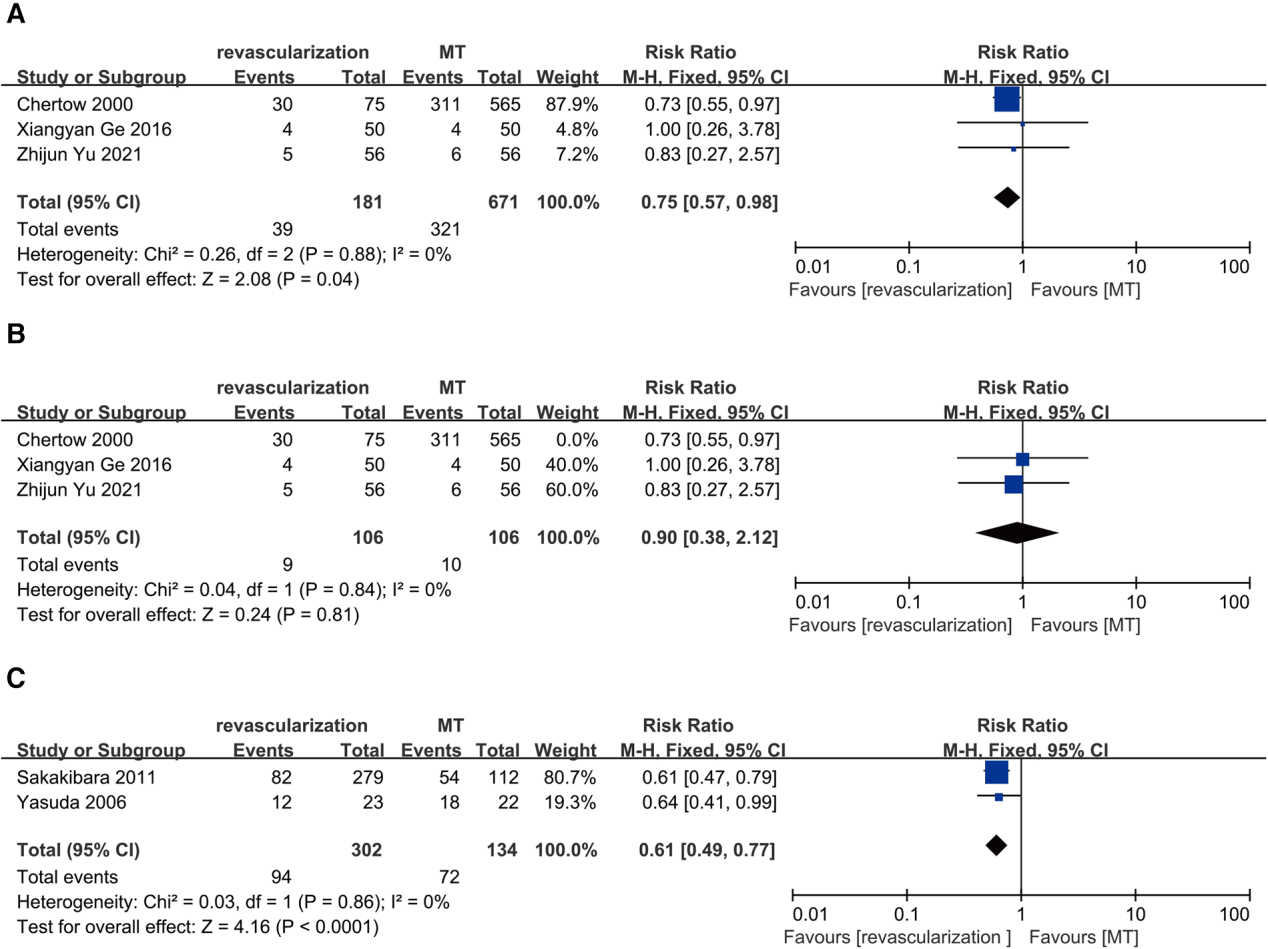
Long-term all-cause death: (**A**) revascularization vs. MT in maintenance dialysis patients with acute coronary syndrome; (**B**) revascularization vs. MT in maintenance dialysis patients with acute coronary syndrome with the exclusion of Chertow 2000. (**C**) revascularization vs. MT in maintenance dialysis patients with single-vessel disease. CI, confidence interval; MT, medical therapy.

Three studies compared revascularization with MT in maintenance dialysis patients with ACS, including 192 patients receiving revascularization and 154 receiving MT. The results showed that revascularization reduces long-term all-cause mortality in maintenance dialysis patients with ACS (RR = 0.75, 95% CI = 0.57–0.98) and no heterogeneity (*I*^2 ^= 0%, *P* = 0.88) (Figure 6A). However, a “leave-one-out” approach for sensitivity analysis found that the exclusion of Chertow 2000 yielded a different result, indicating that revascularization and MT had similar long-term all-cause mortality rates (RR = 0.90, 95% CI = 0.38–2.12) (Figure 6B).

#### 3.6.4. Stable CAD

Meta-analysis was not available for stable CAD because only one study (APPROACH 2018) reported the outcome of maintenance dialysis patients with the condition. The APPROACH study with 118 patients is a prospective cohort study with a follow-up for 8 years. The study comprised 74 patients receiving revascularization (35 receiving PCI and 39 receiving CABG) and 44 receiving MT. The results showed that revascularization was connected with low long-term mortality (adjusted hazard ratio 0.29, 95% CI: 0.15–0.55).

#### 3.6.5. Single-vessel disease

Two studies compared revascularization with MT in maintenance dialysis patients with single-vessel disease, including 302 receiving revascularization (PCI) and 134 receiving MT. The results showed that revascularization reduces long-term all-cause mortality in maintenance dialysis patients with single-vessel disease (RR = 0.61, 95% CI = 0.49–0.77) and no heterogeneity (*I*^2 ^= 0%, *P* = 0.86) (Figure 6C).

#### 3.6.6. Multivessel disease

For multivessel disease, meta-analysis was not conducted because only one study (Yasuda et al. 2006) reported the outcome of maintenance dialysis patients with multivessel disease. Yasuda et al. conducted a prospective cohort study, including 134 maintenance dialysis patients with CAD, of whom 89 had multivessel disease [63 patients were treated with PCI (the revascularization strategy of the study was PCI) and 26 with MT]. The follow-up time was 5 years. The primary endpoint was cardiac death, while the secondary endpoint was all-cause death. The findings showed that in the revascularization and the MT groups, the 5-year all-cause survival rates were 48.4% and 21.8%, respectively (*P* = 0.022), indicating that the long-term all-cause survival rate of the revascularization therapy was better than that of MT in maintenance dialysis patients with multivessel coronary disease.

### 3.7. Sensitivity analysis and publication bias

**Figure 7:**
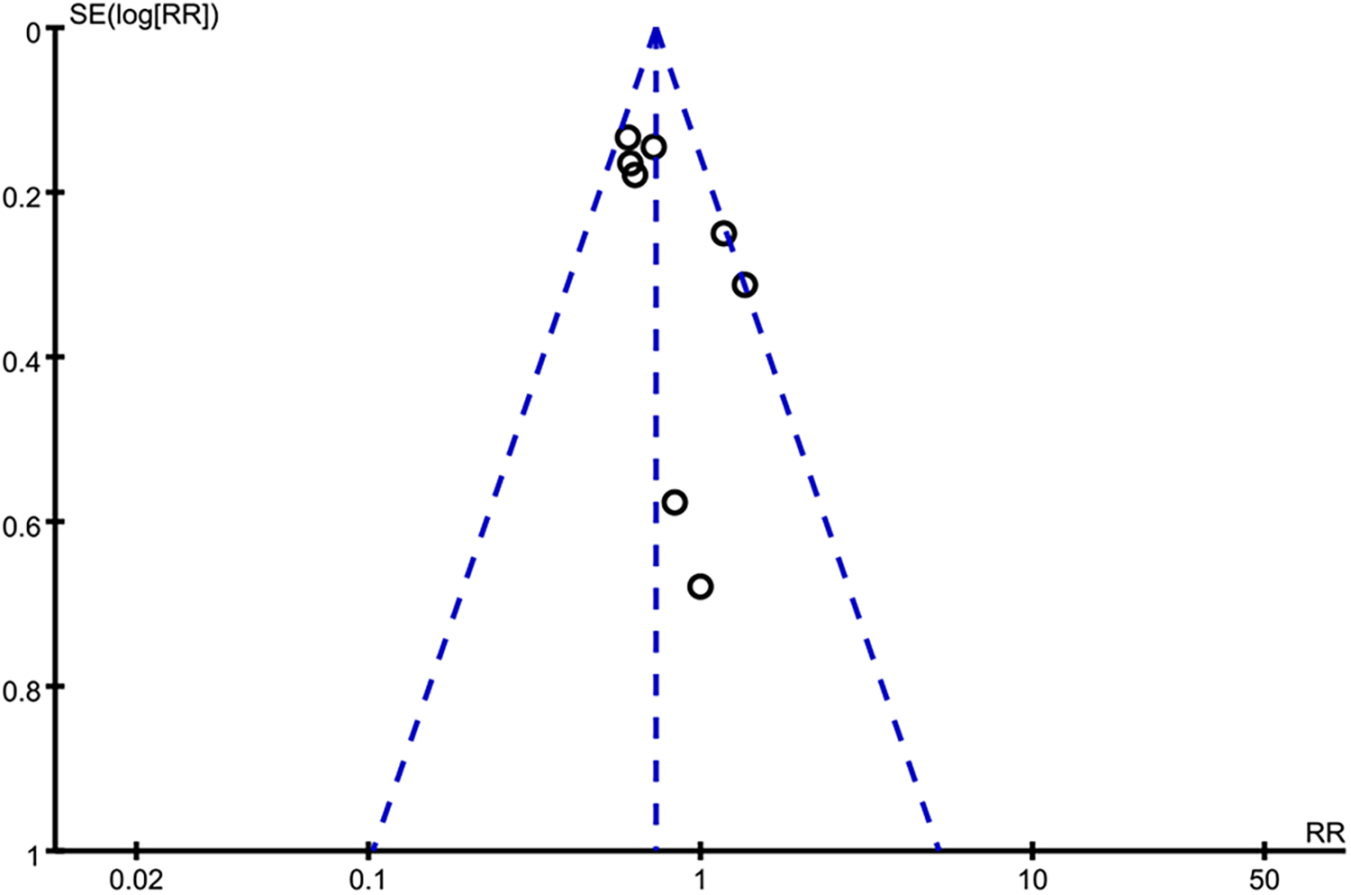
Funnel plot of long-term all-cause mortality. The asymmetrical funnel plot indicates publication bias of this review.

Applying a “leave-one-out” method, we found that excluding anyone did not exert a significant on the result of long-term all-cause death, and long-term cardiac mortality (Supplementary Figures S2, S3). Consistently, excluding anyone did not exert a significant on long-term all-cause death in two subgroups of PCI and CABG (Supplementary Figures S4, S5). For the incidence rate of bleeding events and single-vessel disease, sensitivity analysis was not conducted because only two studies were included. However, for ACS, we found that the exclusion of Chertow 2000 yielded a different result, showing that revascularization and MT had similar long-term all-cause mortality rates (RR = 0.90, 95% CI = 0.38–2.12) (Figure 6B). The funnel plot was asymmetrical (Figure 7), suggesting a publication bias in this meta-analysis.

## 4. Discussion

A total of eight studies with 1,685 patients were selected for this meta-analysis. According to the current findings, revascularization has a lower long-term all-cause mortality and long-term cardiac mortality than MT in maintenance dialysis patients, but a similar incidence rate of bleeding events. Revascularization also showed lower long-term all-cause mortality compared to MT in stable CAD, single-vessel disease, and multivessel disease but did not reduce the long-term all-cause mortality in ACS.

Most previous meta-analyses compared revascularization therapy and MT in patients with CKD and did not isolate the dialysis patients. Only subgroup analysis suggested that revascularization therapy was superior to MT among maintenance patients with CAD (11). However, no meta-analysis specifically compared revascularization therapy and MT among maintenance dialysis patients with CAD. Hence, the present study was an innovation compared to previous meta-analyses because our study only included maintenance dialysis patients.

The results showed that revascularization reduces long-term all-cause mortality among maintenance dialysis patients. When we distinguished the strategy of revascularization, we found different results. PCI decreased the long-term all-cause mortality compared to MT while CABG did not significantly differ from MT. The Yong et al. meta-analysis (21) suggested that PCI had low short-term, medium-term, and long-term all-cause mortality compared with MT, but CABG did not reduce all-cause mortality. However, The Liao et al. meta-analysis (11) showed that PCI and CABG were both associated with lower mortality. It should be noted that the Liao et al. meta-analysis included patients with CKD rather than dialysis patients. CAD in dialysis patients was characterized by multiple-vessel disease, including diffuse vessel disease, small vessel disease, calcification, and left main coronary artery. Another study reported that calcified nodule (CN) was frequently (about 60%) detected by optical coherence tomography (OCT) in CAD patients on dialysis (22). CN is one of the plaque characteristics of patients with ACS or sudden cardiac deaths and associated with major adverse cardiovascular events (MACEs) after PCI (23). All these characteristics led to great obstacles in the process of PCI, particularly in the era of percutaneous transluminal coronary angioplasty and bare metal stent, and usually resulted in failure or inadequate post-expansion following stenting. Modern technology has provided new tools to treat these calcified lesions, including cutting balloons, rotation, laser ablation, and intravascular lithotripsy (IVL) (24), which helps in post-expansion after stent installation. All these developments can improve the prognosis of such patients and change their choice of revascularization. Moreover, the risks of surgical complications in dialysis patients may affect the relative benefit of CABG (25).

When we distinguished the type of CAD, we also found different results. For patients with stable CAD, our results suggested that revascularization was connected with low long-term mortality, which was in contrast to the ISCHEMIA-CKD study. ISCHEMIA-CKD study, the only RCT to date involve large numbers of dialysis patients, suggested that invasive strategy (PCI and CABG) compared to the conservative strategy does not reduce the risk of death or nonfatal myocardial infarction among patients with advanced CKD (more than half are dialysis patients) and stable CAD. Regrettably, the RCT was not included in our meta-analysis because it did not provide the data on the outcomes of dialysis patients treated with revascularization and MT. Among the general population with stable CAD, the COURAGE trial (6) showed no benefit of PCI when compared with MT alone in reducing the risk of death, the BARI-2D trial (26) revealed no significant difference in mortality between revascularization and MT alone, and a meta-analysis of 7 RCTs (27) suggested that PCI was not associated with reducing all-cause mortality. More RCTs are expected to provide evidence of the optimal treatment strategy for dialysis patients in the future. For patients with ACS, we found that revascularization reduces long-term all-cause mortality. However, a different result was yielded when we excluded Chertow 2000. Except Chertow 2000, other qualified studies in this subgroup analysis showed that revascularization fails to reduce long-term all-cause mortality compared to MT. Since RCTs provide a higher level of evidence than cohort studies, we put forth that revascularization fails to reduce long-term all-cause mortality in dialysis patients with ACS. Nonetheless, the conclusion should be explained cautiously. The Yong et al. meta-analysis (21) suggested that PCI does not lower the medium-term all-cause (1 month–1 year) mortality for patients with AMI compared to MT (OR: 0.70; 95% CI: 0.42–1.15, *P* = 0.157). Studies by Medi et al. (28), Chan et al. (29), and Szummer et al. (30) suggested that for patients with ESRD accompanied by NSTEMI and STEMI, PCI does not reduce the risk of death and AMI. However, for non-AMI patients, it can reduce the risk of medium-term MACEs and death for >3 years. Notably, patients with ACS in the two single-center small samples of RCTs presented unstable angina, and AMI patients accounted for a small proportion of 6% in the study by Gen (2016) study and 17% in Yu (2021). The revascularization strategy of both RCTs was PCI, and the follow-up duration was only 1 year. Intriguingly, if more AMI patients can be included and the follow-up time can be prolonged, a different conclusion may be reached.

In addition, the results of long-term cardiac mortality and the incidence of bleeding events analyses demonstrated that revascularization reduces long-term cardiac mortality without increasing the incidence of bleeding events. Navarese et al. meta-analysis (31) demonstrated, for the first time, that in stable CAD patients, revascularization yielded a lower risk of cardiac death compared to MT, and the benefit of revascularization on cardiac survival increased gradually over time, with a 19% decline in RR for each additional 4 years of follow-up. The enduring advantage of revascularization as opposed to attenuation of medical adherence over time, less spontaneous MI, and a temporal attenuation of post-revascularization early procedure complications may be viable explanations for increasing cardiac survival benefits post-prolonged follow-ups. Typically, maintenance hemodialysis patients have a high risk of bleeding due to platelet dysfunction and alterations in the interaction between platelets and artery walls (32). DAPT should be implemented for 12 months following PCI rather than 6 months in patients with a high risk of bleeding, according to a most recent study. The risk of bleeding is increased when DAPT is administered to patients with renal insufficiency who already have platelet dysfunction (33–35). Several studies have demonstrated that the risk of bleeding does not rise in hemodialysis patients treated with an antiplatelet medication (36), while some studies have shown that the bleeding risk increases (37). Another meta-analysis revealed that hemodialysis patients on two antiplatelet medications had an increased risk of bleeding, while those taking just one antiplatelet medication did not face a similar risk (38). Our meta-analysis results suggested that the DAPT after PCI in dialysis patients does not increase the risk of bleeding. However, these two studies are single-center small-sample studies with a short follow-up time; hence, the results have some limitations. The clinical use of antiplatelet drugs in such patients should be cautious and needs to be investigated further.

For multivessel disease, Yasuda et al. suggested that revascularization was linked with low long-term mortality. Consistently, the single-vessel disease subgroup also reached a similar conclusion. Many RCTs on patients with CAD in the general population have suggested that PCI does not increase the survival rate for those with single-vessel disease but that it was connected with higher cardiac survival compared to MT in those with two-vessel disease. Additionally, CABG was connected with a better survival compared to PCI in those with three-vessel disease (14). However, our results suggested that revascularization shows a survival benefit over MT among dialysis patients with CAD independent of the number of diseased arteries. Reportedly, 70% of dialysis patients had left ventricular hypertrophy (LVH) caused by continuous hemodynamic overload state. LVH reduces coronary reserve and causes severe LV dysfunction, and uremia-related risk factors and traditional risk factors accelerate arteriosclerosis among dialysis patients (39). Considering the distinctive clinical characteristics of dialysis patients, revascularization may be required for single-vessel disease in maintenance dialysis patients.

Furthermore, the best drug treatment scheme for CAD in dialysis patients is not yet clarified, and the results of existing studies will be affected by the drug treatment scheme. For example, antiplatelet therapy with drugs was a cornerstone of CAD. Some studies have shown that the risk of MI in dialysis patients treated with antiplatelet therapy is significantly reduced, but total mortality does not alter appreciably as a result (40). DAPT with clopidogrel plus aspirin has been associated with a lower rate of MACE in patients after PCI than aspirin monotherapy (41). According to some observational studies, persistently high platelet reactivity despite antiplatelet medication is linked to a high risk of definite or probable stent thrombosis, nonfatal MI, and cardiovascular death (42). Diabetes mellitus (DM), an independent predictor of clinical outcomes following PCI in dialysis patients, should also be considered as one of the factors affecting the incidence of events. DM is currently regarded as the primary cause of ESKD in western countries (43), with a percentage of affected patients that range from 30% in European region to 45% in USA (44). A retrospective study including 274 dialysis patients who underwent PCI suggested that mortality and MACE were increased two-fold in the presence of DM (45).

## 5. Limitations

Firstly, most of the eight articles included in this meta-analysis were nonrandomized studies; hence, selection and confounding biases were unavoidable. Secondly, due to the scarcity of data, we were unable to obtain the specific details of drug treatment, stent type used, type of ACS and diabetes patients' clinical outcomes. The conclusion would be more convincing if subgroup analyses were conducted based on these variables. Thirdly, we did not account for the follow-up time variations. Previous studies have shown that a strong and consistent reduction of cardiac mortality in favor of revascularization is directly associated with the duration of follow-up (31). Fourthly, only Chinese and English articles were included in this study, which may lead to language bias. Finally, this study might have publication bias.

## 6. Conclusion

Long-term all-cause mortality and long-term cardiac mortality were reduced by revascularization in comparison to MT alone in patients undergoing dialysis. However, biases are unavoidable and the generalizability of the results is affected when comparing revascularization strategies to MT alone, because MT is not specified. Larger RCTs are needed to confirm the conclusion of this meta-analysis.

## Data Availability

The original contributions presented in the study are included in the article/**Supplementary Material**, further inquiries can be directed to the corresponding authors.
