# Supplementary material for: Comparison of revascularization with conservative medical treatment in maintenance dialysis patient with coronary artery disease: a systemic review and meta-analysis
Source: Front Cardiovasc Med. 2023 Apr 17;10:1143895. doi: 10.3389/fcvm.2023.1143895 (PMC10149751; doi:10.3389/fcvm.2023.1143895)
Supplement: Supplementary file 1 [file Datasheet1.docx]

***Supplementary Material***

**Words Search Strategy:**

**Cochrane Library database**


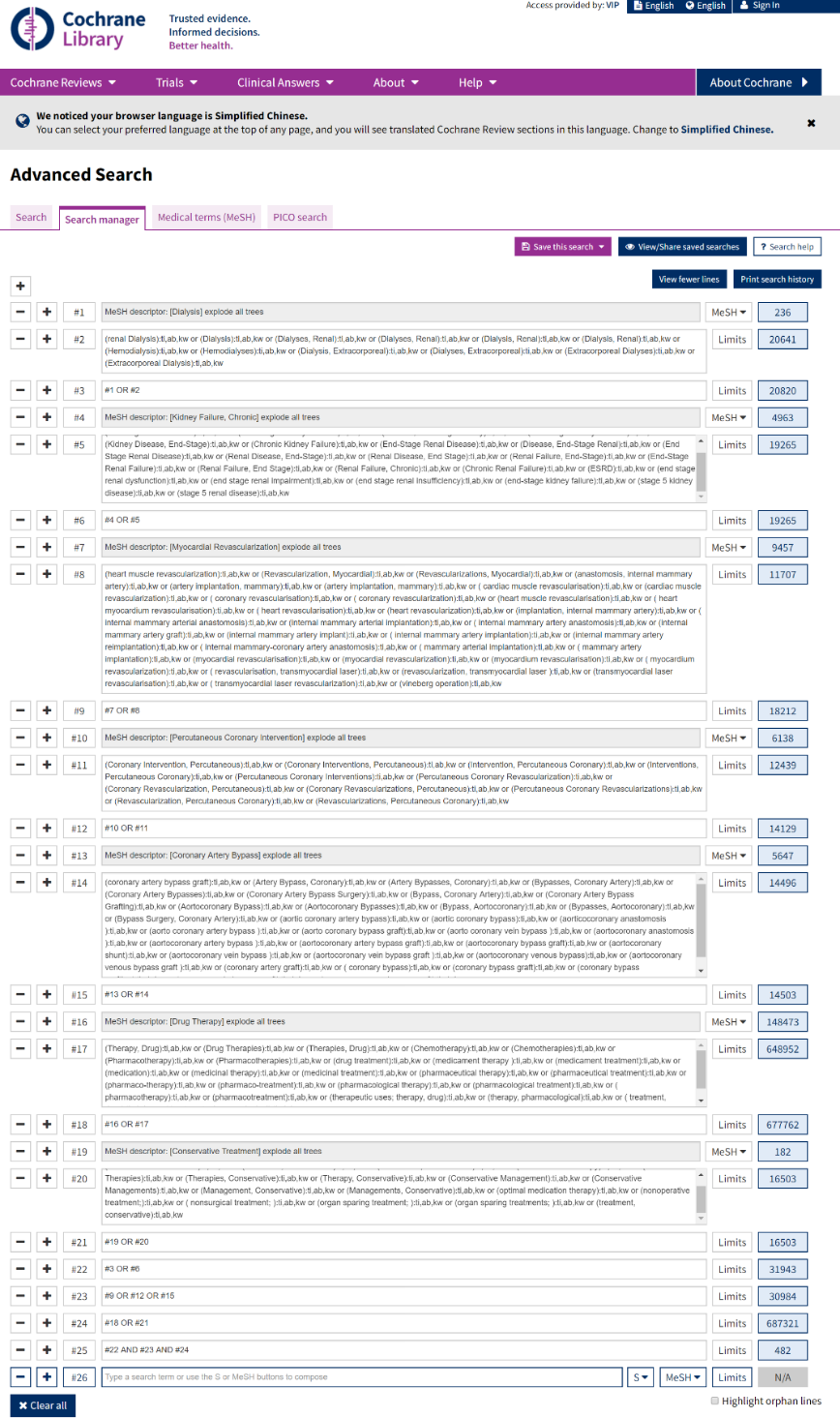


FIGURE 1 | Search Strategy of Cochrane Library database

Cochrane Library database

**PubMed（2022-10-12）：**Search: (((("Renal Dialysis"[Mesh]) OR (((((((((Dialysis[Title/Abstract]) OR (Dialyses, Renal[Title/Abstract])) OR (Dialyses, Renal[Title/Abstract])) OR (Dialysis, Renal[Title/Abstract])) OR (Hemodialysis[Title/Abstract])) OR (Hemodialyses[Title/Abstract])) OR (Dialyses, Extracorporeal[Title/Abstract])) OR (Extracorporeal Dialyses[Title/Abstract])) OR (Extracorporeal Dialysis[Title/Abstract]))) OR (("Kidney Failure, Chronic"[Mesh]) OR (((((((((((((((((((((((End-Stage Kidney Disease[Title/Abstract]) OR (Disease, End-Stage Kidney[Title/Abstract])) OR (End Stage Kidney Disease[Title/Abstract])) OR (Kidney Disease, End-Stage[Title/Abstract])) OR (Chronic Kidney Failure[Title/Abstract])) OR (End-Stage Renal Disease[Title/Abstract])) OR (Disease, End-Stage Renal[Title/Abstract])) OR (End Stage Renal Disease[Title/Abstract])) OR (Renal Disease, End-Stage[Title/Abstract])) OR (Renal Disease, End Stage[Title/Abstract])) OR (Renal Failure, End-Stage[Title/Abstract])) OR (End-Stage Renal Failure[Title/Abstract])) OR (Renal Failure, End Stage[Title/Abstract])) OR (Renal Failure, Chronic[Title/Abstract])) OR (Chronic Renal Failure[Title/Abstract])) OR (ESRD[Title/Abstract])) OR (end stage renal dysfunction[Title/Abstract])) OR (end stage renal impairment[Title/Abstract])) OR (end stage renal insufficiency[Title/Abstract])) OR (end-stage kidney failure[Title/Abstract])) OR (stage 5 kidney disease[Title/Abstract])) OR (stage 5 renal disease[Title/Abstract])) OR (end stage renal disease[Title/Abstract])))) AND (((("Myocardial Revascularization"[Mesh]) OR ((((((((((((((((((((((((((((((((Revascularization, Myocardial[Title/Abstract]) OR (Revascularizations, Myocardial[Title/Abstract])) OR (anastomosis, internal mammary artery[Title/Abstract])) OR (artery implantation, mammary[Title/Abstract])) OR (cardiac muscle revascularisation[Title/Abstract])) OR (cardiac muscle revascularization[Title/Abstract])) OR (coronary revascularisation[Title/Abstract])) OR (coronary revascularization[Title/Abstract])) OR (heart muscle revascularisation[Title/Abstract])) OR (heart myocardium revascularisation[Title/Abstract])) OR (heart revascularisation[Title/Abstract])) OR (heart revascularization[Title/Abstract])) OR (implantation, internal mammary artery[Title/Abstract])) OR (internal mammary arterial anastomosis[Title/Abstract])) OR (internal mammary arterial implantation[Title/Abstract])) OR (internal mammary artery anastomosis[Title/Abstract])) OR (internal mammary artery graft[Title/Abstract])) OR (internal mammary artery implant[Title/Abstract])) OR (internal mammary artery implantation[Title/Abstract])) OR (internal mammary artery reimplantation[Title/Abstract])) OR (internal mammary-coronary artery anastomosis[Title/Abstract])) OR (mammary arterial implantation[Title/Abstract])) OR (mammary artery implantation[Title/Abstract])) OR (myocardial revascularisation[Title/Abstract])) OR (myocardial revascularization[Title/Abstract])) OR (myocardium revascularisation[Title/Abstract])) OR (myocardium revascularization[Title/Abstract])) OR (revascularisation, transmyocardial laser[Title/Abstract])) OR (revascularization, transmyocardial laser[Title/Abstract])) OR (transmyocardial laser revascularisation[Title/Abstract])) OR (transmyocardial laser revascularization[Title/Abstract])) OR (vineberg operation[Title/Abstract]))) OR (("Percutaneous Coronary Intervention"[Mesh]) OR (((((((((((Coronary Intervention, Percutaneous[Title/Abstract]) OR (Coronary Interventions, Percutaneous[Title/Abstract])) OR (Intervention, Percutaneous Coronary[Title/Abstract])) OR (Interventions, Percutaneous Coronary[Title/Abstract])) OR (Percutaneous Coronary Interventions[Title/Abstract])) OR (Percutaneous Coronary Revascularization[Title/Abstract])) OR (Coronary Revascularization, Percutaneous[Title/Abstract])) OR (Coronary Revascularizations, Percutaneous[Title/Abstract])) OR (Percutaneous Coronary Revascularizations[Title/Abstract])) OR (Revascularization, Percutaneous Coronary[Title/Abstract])) OR (Revascularizations, Percutaneous Coronary[Title/Abstract])))) OR (("Coronary Artery Bypass"[Mesh]) OR (((((((((((((((((((((((((((((((((Artery Bypass, Coronary[Title/Abstract]) OR (Artery Bypasses, Coronary[Title/Abstract])) OR (Bypasses, Coronary Artery[Title/Abstract])) OR (Coronary Artery Bypasses[Title/Abstract])) OR (Coronary Artery Bypass Surgery[Title/Abstract])) OR (Bypass, Coronary Artery[Title/Abstract])) OR (Coronary Artery Bypass Grafting[Title/Abstract])) OR (Aortocoronary Bypass[Title/Abstract])) OR (Aortocoronary Bypasses[Title/Abstract])) OR (Bypass, Aortocoronary[Title/Abstract])) OR (Bypasses, Aortocoronary[Title/Abstract])) OR (Bypass Surgery, Coronary Artery[Title/Abstract])) OR (aortic coronary artery bypass[Title/Abstract])) OR (aortic coronary bypass[Title/Abstract])) OR (aorticocoronary anastomosis[Title/Abstract])) OR (aorto coronary artery bypass[Title/Abstract])) OR (aorto coronary bypass graft[Title/Abstract])) OR (aorto coronary vein bypass[Title/Abstract])) OR (aortocoronary anastomosis[Title/Abstract])) OR (aortocoronary artery bypass[Title/Abstract])) OR (aortocoronary artery bypass graft[Title/Abstract])) OR (aortocoronary bypass graft[Title/Abstract])) OR (aortocoronary shunt[Title/Abstract])) OR (aortocoronary vein bypass[Title/Abstract])) OR (aortocoronary vein bypass graft[Title/Abstract])) OR (aortocoronary venous bypass[Title/Abstract])) OR (aortocoronary venous bypass graft[Title/Abstract])) OR (coronary artery graft[Title/Abstract])) OR (coronary bypass[Title/Abstract])) OR (coronary bypass graft[Title/Abstract])) OR (coronary bypass grafting[Title/Abstract])) OR (coronary vein bypass graft[Title/Abstract])) OR (coronary venous bypass graft[Title/Abstract]))))) AND ((("Drug Therapy"[Mesh]) OR (((((((((((((((((((((((((Therapy, Drug[Title/Abstract]) OR (Drug Therapies[Title/Abstract])) OR (Therapies, Drug[Title/Abstract])) OR (Chemotherapy[Title/Abstract])) OR (Chemotherapies[Title/Abstract])) OR (Pharmacotherapy[Title/Abstract])) OR (Pharmacotherapies[Title/Abstract])) OR (drug treatment[Title/Abstract])) OR (medicament therapy[Title/Abstract])) OR (medicament treatment[Title/Abstract])) OR (medication[Title/Abstract])) OR (medicinal therapy[Title/Abstract])) OR (medicinal treatment[Title/Abstract])) OR (pharmaceutical therapy[Title/Abstract])) OR (pharmaceutical treatment[Title/Abstract])) OR (pharmaco-therapy[Title/Abstract])) OR (pharmaco-treatment[Title/Abstract])) OR (pharmacological therapy[Title/Abstract])) OR (pharmacological treatment[Title/Abstract])) OR (pharmacotherapy[Title/Abstract])) OR (pharmacotreatment[Title/Abstract])) OR (therapeutic uses; therapy, drug[Title/Abstract])) OR (therapy, pharmacological[Title/Abstract])) OR (treatment, drug[Title/Abstract])) OR (treatment, pharmacological[Title/Abstract]))) OR (("Conservative Treatment"[Mesh]) OR (((((((((((((((((Conservative Treatments[Title/Abstract]) OR (Treatment, Conservative[Title/Abstract])) OR (Treatments, Conservative[Title/Abstract])) OR (Conservative Therapy[Title/Abstract])) OR (Conservative Therapies[Title/Abstract])) OR (Therapies, Conservative[Title/Abstract])) OR (Therapy, Conservative[Title/Abstract])) OR (Conservative Management[Title/Abstract])) OR (Conservative Managements[Title/Abstract])) OR (Management, Conservative[Title/Abstract])) OR (Managements, Conservative[Title/Abstract])) OR (optimal medication therapy[Title/Abstract])) OR (nonoperative treatment;[Title/Abstract])) OR (nonsurgical treatment;[Title/Abstract])) OR (organ sparing treatment;[Title/Abstract])) OR (organ sparing treatments;[Title/Abstract])) OR (treatment, conservative[Title/Abstract]))))

**Embase:**

**
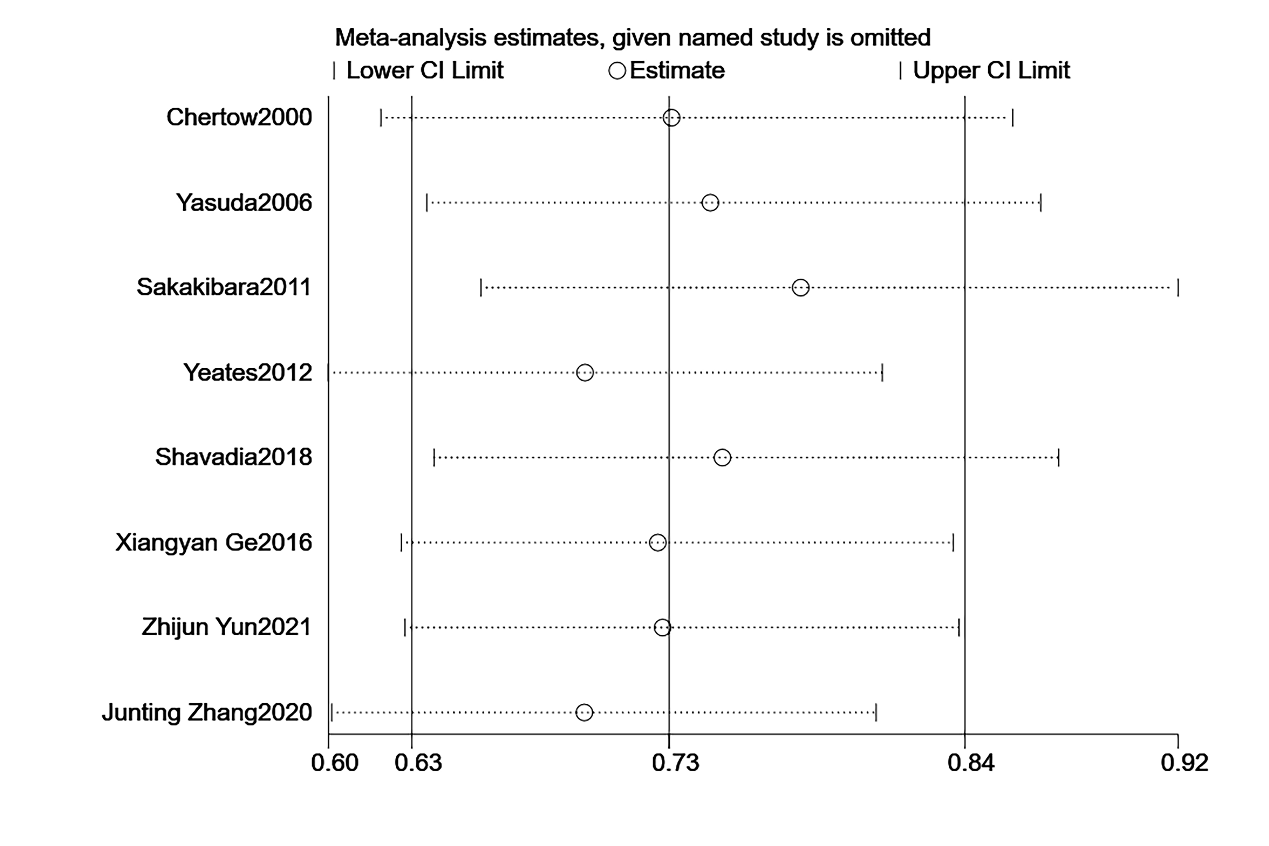
**

FIGURE 2 | Sensitivity analysis of long-term all-cause death

**
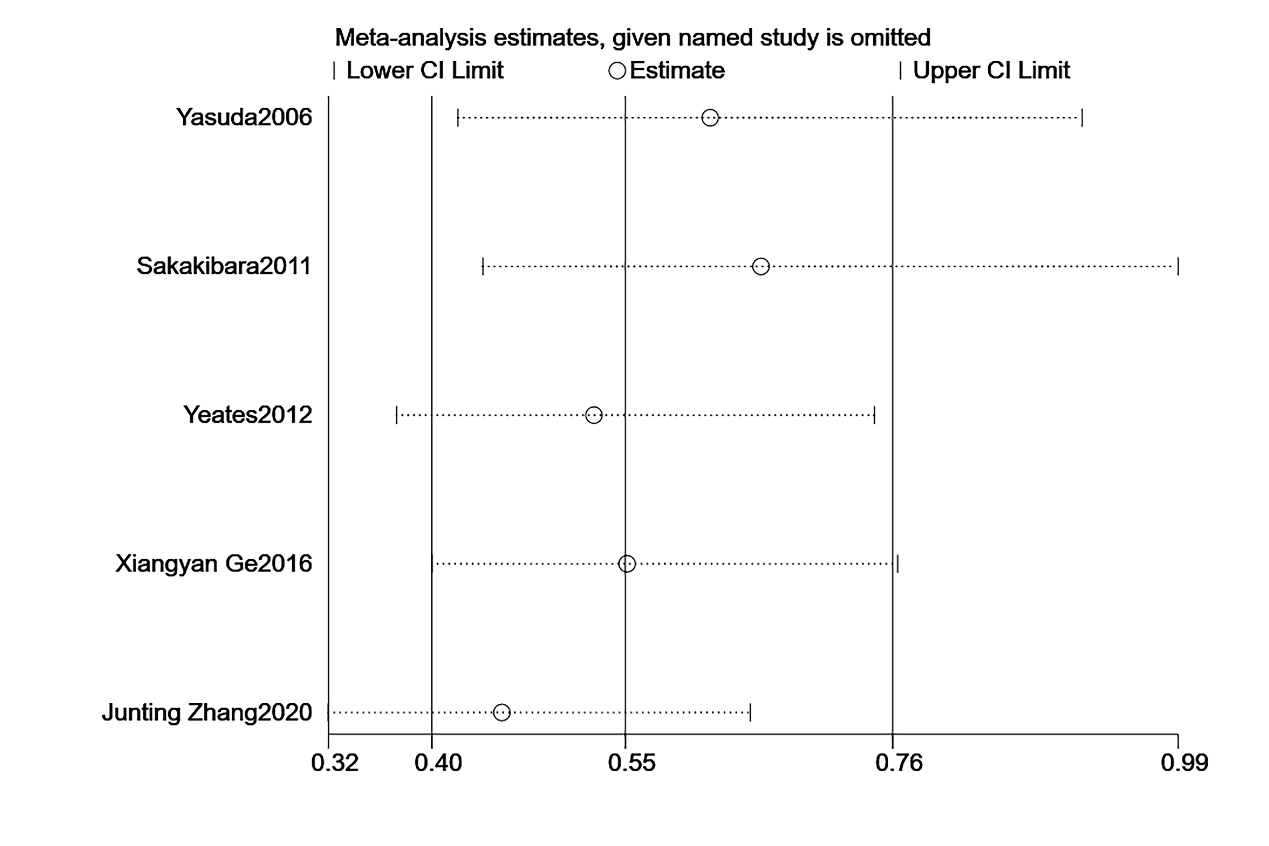
**

FIGURE 3 | Sensitivity analysis of long-term cardiac mortality

**
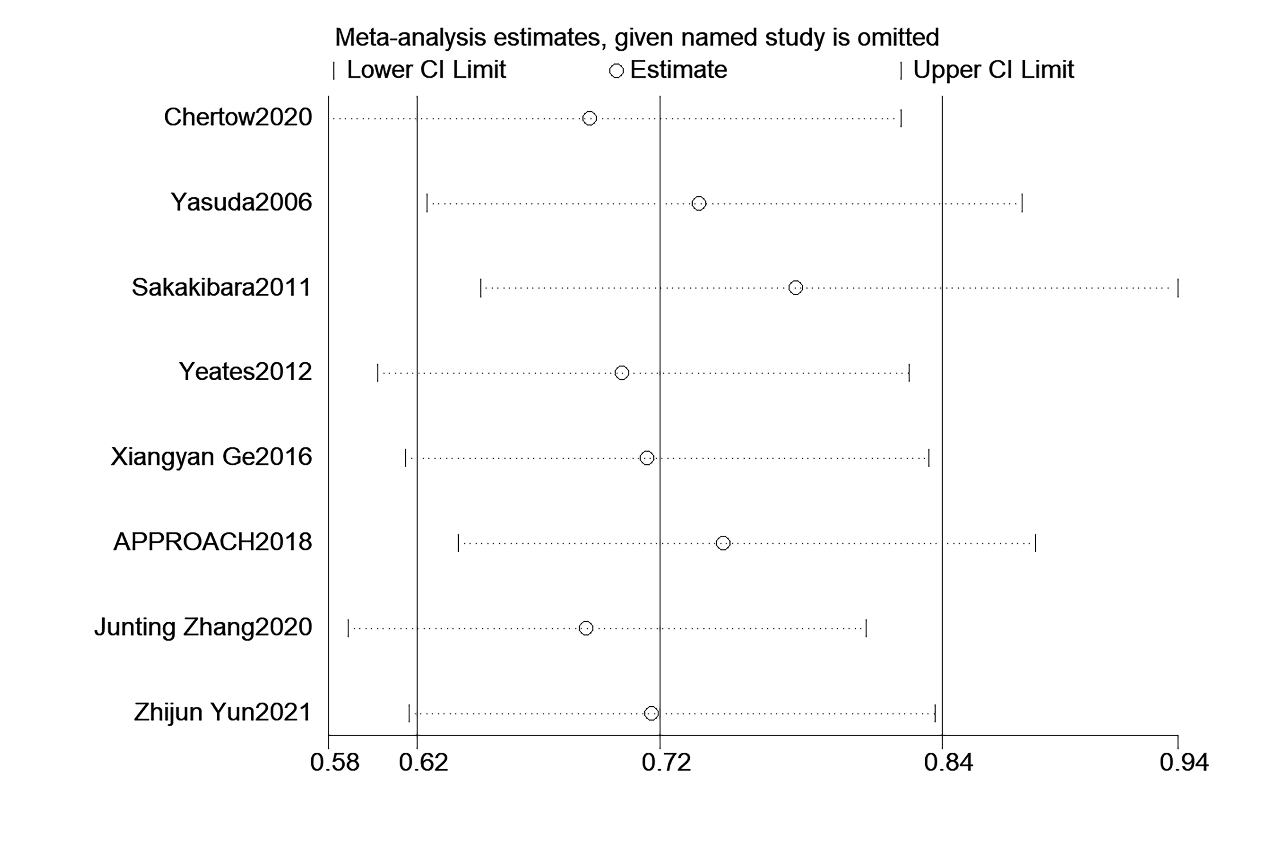
**

FIGURE 4 | Sensitivity analysis of long-term all-cause death in subgroup of PCI

**
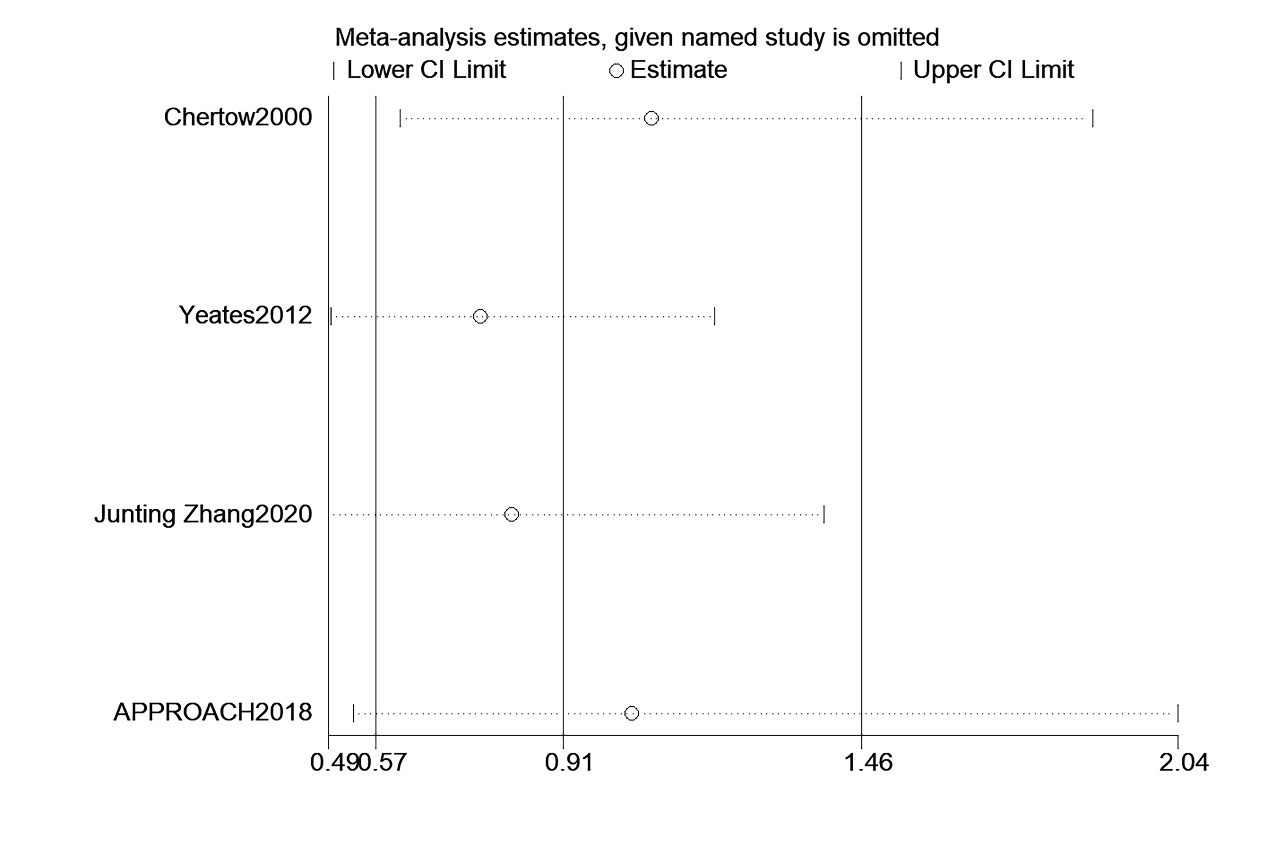
**

FIGURE 5 | Sensitivity analysis of long-term all-cause death in subgroup of CABG
